# Synchronized Incidental Affect Changes Ambiguity Preferences

**DOI:** 10.21203/rs.3.rs-3873970/v1

**Published:** 2024-01-31

**Authors:** Deshawn Sambrano, Bryan Dong, Paul Glimcher, Elizabeth A. Phelps

## Abstract

Decisions under uncertainty are prevalent, but come under two distinct types. Risk, which has unknown outcomes but known probabilities for those outcomes and ambiguity which contains both unknown outcomes and unknown probabilities. Although there have been several studies linking affect and aversion to ambiguity, there have been no studies that have to identify how changing one’s affective response can change their choices. A total of 166 adults ( *M* = 36.54, *SD* = 11.80) participated in an online study through Prolific. Participants were presented with a lottery on each trial which varied on its uncertainty type (risky vs ambiguous) and winning characteristics (winning probability and amount). Half of the ambiguous lotteries were paired with an neutral image (e.g., office supplies), while the other half was paired with an emotionally evocative image (e.g., burning house) that was hypothesized to incidentally influence their decisions. As measured by both raw choice data as well as through a computational model, participants were more averse to ambiguity when the lottery was paired with an emotionally evocative image. Follow-up analyses revealed that only lotteries in which the computational model predicted the participant would choose the lottery were affected by the images. This study highlights the phenomenon in which one’s awareness of an affective stimulus can alter its impact on their decisions.

### Full Text

Our life is the sum total of the decisions we make. As such, it is prudent that we improve our decisions in any way we can by identifying potential biases that can lead us to make worse decisions. One prolific bias in decision making is ambiguity aversion. Ambiguous decisions have both *unknown* outcomes and *unknown* probabilities. In contrast, risky decisions have an *unknown* outcome, but *known* probabilities for those potential outcomes. People tend to avoid ambiguous options more so than risky ones (Ellsberg, 1961), even when the risky option has a higher expected value (FeldmanHall et al., 2016; Levy et al., 2010; Raio et al., 2022; Sambrano et al., 2022). Crucially, this bias against ambiguity can lead to poor outcomes in a variety of decisions, including health (Han et al., 2009), social (Li et al., 2020), and career decisions (Xu, 2020). The current study attempts to understand what affects ambiguity aversion and whether this preference can be shifted.

Ambiguity research has shown a clear link with affect. Specifically, neuroimaging and psychophysiology studies have shown that viewing ambiguous lotteries elicits an affective response that can be used to predict choices. An imaging study found that the blood oxygen level dependent (BOLD) signal in the amygdala (a region commonly involved in processing affect) is greater for ambiguous versus risky choices (Levy et al., 2010). Another study found that skin conductance responses to viewing lotteries can be used to predict ambiguous, but not risky decisions (FeldmanHall et al., 2016). Both studies measured an affective response that was integral to ambiguous choices, in other words the affective response was elicited by the choice itself.

In contrast, we can also manipulate affect independent of choice—typically called incidental affect. In an incidental affect and decision making study, an affective state is induced and *then* participants make a series of decisions. The affective state is unrelated to the choices and should be ignored; however, incidental affect often alters one's choices (Lerner et al., 2015; Phelps et al., 2014). In the context of ambiguity aversion, a recent study attempted to influence ambiguity preferences with two incidental affect manipulations (Sambrano et al., 2022). The first used the Trier Social Stress Test to induce a stress response, after which participants completed a series of risky and ambiguous lotteries. The second used transient threats of electric shock to incidentally induce an arousal response. Neither manipulation altered ambiguity or risk preferences. Based on these findings, it was hypothesized that incidental stress and arousal did not affect ambiguity preferences because participants were able to disentangle the manipulation evoked affective response from the affect induced by the choices themselves. In sum, ambiguity preferences appear to be influenced by an integral affective response to the choice, but are generally resilient to acute, incidental affect manipulations administered prior to the choice.

With regard to ambiguity, people are able to dissociate an arousal or stress response evoked immediately prior to the decision task from an arousal response evoked by the uncertain choice option in a variety of contexts. However, other studies exploring different affective responses have shown evidence of incidental affect on risky and ambiguous decisions. Raio et al. (2022) found that stressors accumulated over the course of one's life predicted ambiguity attitudes, such that individuals with more life stress were more averse to ambiguity. They concluded that a history of stressful events can lead a person to avoid uncertain options, essentially expecting the worst outcome. In the Raio et al. (2022) study, stressors occurred long before the participants made decisions in the experiment and were not induced by the experimenter. It is unlikely that participants were reflecting on their stressful life histories when performing that laboratory decision making task, but nonetheless, those affective experiences incidentally impacted their preferences.

Judgements of ambiguous stimuli have also been shown to be influenced by integral and incidental affect. In these studies, participants were presented with an ambiguous image, such as a face expressing surprise, along with non ambiguous faces (i.e., happy and angry expressions). The participants' task was to evaluate the valence of each face. These experiments have also shown that integral affective responses are associated with the evaluation of ambiguous stimuli. Specifically, increased activity in the amygdala (Kim et al., 2003), higher levels of salivary cortisol (Brown et al., 2017), and trait anxiety (Park et al., 2016) were associated with a negativity bias in judging the valence of ambiguous facial expressions. These types of studies have also shown a potential link between incidental arousal and ambiguous judgements. For example, threats of shock lead to increased negativity bias for judgements of ambiguous stimuli (Neta et al., 2017).

Although there is some evidence that acute, incidental affect manipulations can influence judgements for some types of ambiguous stimuli, there is no evidence they affect ambiguity preferences in an incentive compatible monetary decision making task. It is possible that previous attempts to show an impact of acute, incidental arousal on ambiguity preferences in this type of task were unsuccessful because participants were able to dissociate the affective response to the manipulation from the affective response driven by the choice. In spite of the fact that the arousal or stress response may be present during the choice, if the participant can clearly identify that the source of the arousal or stress response is independent of the choice, they may be able to reduce or eliminate its impact on the decision. This interpretation would be consistent with classic studies by Schachter and Singer (1962) and Schwarz and Clore (1983), which highlight how internal (pharmacological) and external (weather) affect manipulations can influence judgements, when the potential effects are not clear. Taken together, it appears that people tend to treat incidental factors as reliable information when making judgements unless prompted with information to suggest otherwise.

In the present study, we examined if an acute and relatively mild incidental arousal manipulation would shift ambiguity preferences if it was more difficult to disentangle from the integral affective response to the choices itself. To achieve this, we induced an incidental affective response that was temporally correlated with the choice with emotionally evocative images. Although image viewing is putatively a weaker affect manipulation than psychosocial stress or threats of shock, it may be difficult to separate the source of the affective response when the affective stimulus occurs simultaneously with a choice. Studies on visual attention have shown that processing emotional stimuli is automatic and uncontrollable (Anderson & Phelps, 2001; Phelps et al., 2006). As such, we might expect that incidental, arousing stimuli synchronized with the choice to be processed automatically and simultaneously with a choice option, making it difficult to differentiate the affective response *integral* to the choice and the *incidental* response evoked by viewing the image. If so, we would expect participants to show different ambiguity preferences for choices paired with emotionally evocative images compared to control images.

### Methods

Based on an *a priori* power analysis, we planned a sample size of 170 subjects. We recruited 200 participants using Prolific. We filtered for people that lived inside the United States, had a history of completed studies (<95% with more than 100 attempts), and were fluent in English. Additionally, we included a variety of checks in the experiment to ensure quality data (see *Data Preparation and Screening*). We excluded participants who failed attention check questions and had poor data quality leaving a total of 166 participants available for data analysis (50% Female; $M_{Age}$ = 36.54, $SD_{Age}$ = 11.80).

## Measures

We adapted an incentive compatible risky/ambiguous lottery task (FeldmanHall et al., 2016; Levy et al., 2010; Raio et al., 2022; Sambrano et al., 2022) to elicit an affective response synchronized with the onset of the choice options. Participants saw a lottery on screen and decided whether they would prefer a certain $5 or to play that lottery to potentially earn more money. Each lottery had two potential outcomes: winning and losing. Lottery winnings ranged from $5 to $66, while the losing outcome resulted in $0. There were two types of lotteries: risky and ambiguous. On risky lotteries, participants had complete outcome distribution information, and risky lotteries had three winning probabilities ($p$ = .25, .5, .75).

During ambiguous lotteries, the outcome distributions were partially occluded; there were three levels of ambiguity covering varying portions of the outcome distribution (*A* = .24%, %.5%, .74%). For all ambiguous lotteries, the true winning probability was set to .50.

Participants were told lotteries consisted of 100 red and blue chips, which indicated the probability of that outcome. Lotteries were visualized with a vertical rectangle where the proportion colored red and blue, and the corresponding number in each colored section, represented the probability of that outcome occurring. On ambiguous lotteries, a portion of the colored sections, representing the outcome probabilities, was occluded by an image. For example, a lottery with 50% ambiguity, would show 25% red and 25% blue, and the remaining 50% would be occluded with the image (see Fig. 1a). Participants were informed that the section of the rectangle occluded image could be all red, all blue, or some combination of red and blue. However, to control for risk preferences, the *objective* winning probability for all ambiguous lotteries was .50, such that exactly, half of the occluded section was red and half was blue. Outcomes associated with each color were presented above and below the lottery rectangles.

The main difference between this task and previous iterations (FeldmanHall et al., 2016; Levy et al., 2010; Raio et al., 2022; Sambrano et al., 2022) was the use of an emotionally charged or neutral image as the occluder (see Fig. 1). These images were selected to evoke an affective response that was synchronized with the choice onset and offset. As a result of the synchronization, we hypothesized that the affective response to the image would be more difficult to dissociate from the lottery induced affective response and be unintentionally incorporated into the lottery's utility function. Using images as the occluders led to one additional difference from previous iterations. In previous versions of the task, the occluder changed size to correspond to the proportion of the lottery's distribution being covered. One consequence of using images as an occluder is that the occluder could not change size without distorting the image or dramatically changing the amount of space the occluder covered. To account for this, the occluding images' size remained constant and instead the distributions (represented by the red and blue rectangles) changed size to ensure the relative visible proportions of each stimuli matched their task probabilities.

Occluding images were selected from the Open Affective Standardized Image Set (OASIS; Kurdi et al., 2017). This database provides many benefits over commonly used image databases such as International Affective Picture System (IAPS; Lang, 2005) because these images are more modern, are more accessible to researchers, and OASIS provides additional data on the participants rating the images. OASIS contains 900 images from a variety of different categories. Each image provides a standardized valence (1 = *Very Negative* to 7 = *Very Positive*) and arousal (1 = *Very Low* to 7 = *Very High*) rating averaged over 822 participants varying on ideology, education, and socioeconomic status (SES). A subset of 20 images were selected as occluders for the lottery task. Ten images were selected specifically such that they had negative valence (*M* = 1.6) and high arousal (*M* = 5.2). A second criterion was placed such that they had approximately equal arousal ratings across genders. Another subset of 10 images with neutral valence (*M* = 4.0) and low arousal (*M* = 1.8) were selected for control trials.

In addition to the main lottery task, participants completed a series of survey questions. First, they rated the valence and arousal of each of the 20 images. Participants were given a detailed description of each term when rating valence and arousal (see Appendix A; adapted from Kurdi et al., 2017). Subjects used a 100 point slider scale (0 =  or to 100 =  or ) for both ratings. In addition to the image ratings, participants also completed a series of standard questionnaires.
Appendix A; adapted from Kurdi et al., 2017). Subjects used a 100 point slider scale (0 = *Low Arousal* or *Very Negative* to 100 = *High Arousal* or *Very Positive*) for both ratings. In addition to the image ratings, participants also completed a series of standard questionnaires. Specifically, they completed: the Positive Affect Negative Affect Scale (PANAS; Watson et al., 1988), the Trait Anxiety Inventory (TAI; Spielberger et al., 1983), the Perceived Stress Scale (PSS; Cohen et al., 1983), and the Intolerance of Uncertainty (IoU) scale (Carleton et al., 2007). These scales were included to assess whether there are any psychological factors which mediate changes in ambiguity preferences as a result of the images.

## Procedures

The experiment was created for an online sample using the jsPsych framework (de Leeuw, 2015). After informed consent, participants viewed the instructions for the lottery task. To ensure they understood the task, participants were required to complete a comprehension check, which included several questions about probabilities described in lottery urns as well as how the bonus was computed. Participants were allowed to correct one mistake on the comprehension check before being removed from the experiment.

Upon completion of the comprehension check, participants began a series of 36 practice trials to become familiar with the task. All occluders during the practice trials used neutral images. For the main lottery task, we used a within subjects design in which participants completed 180 trials organized into two blocks. Sixty trials were risky (full distribution information) and 120 were ambiguous (partially occluded distribution). Participants viewed the ambiguous lotteries twice, once paired with the emotionally evocative images ($N = 60$) and once paired with the neutral images ($N = 60$). All images were preloaded into the computer’s random access memory at the beginning of the experiment to ensure they would load onto the page simultaneously with the lottery. Trials started with lotteries viewable for four seconds. This was followed by a screen with the words “Lottery” and “$5” where participants had 1.5 seconds to implement their decision. Between each trial they saw a fixation cross for one second. Winning amount, winning color, winning probability, and proportion of ambiguity were counterbalanced within subjects to ensure both colors were equated on expected value. The location of the word “Lottery” during the choice implementation phase was counterbalanced across individuals. Lastly, the occluder image was randomly selected for each trial for each individual.

Once finished, subjects completed an attention check question which asked them about the images seen during the experiment and a random trial to be realized as a bonus. Specifically, the bonus was based on their decision on a randomly selected trial. If they chose the reference option on that trial, they would receive $5 as a bonus. In contrast, if they selected the lottery option, we would simulate a draw from that lottery option, where their bonus was determined by the result of this simulated draw. Finally, participants were then directed to a Qualtrics survey where they completed all questionnaires, answered basic demographic questions, and were informed of their bonus payment.

## Analytic Plan

We measured the effects of emotionally evocative images on ambiguity preferences with two methods: raw choice data and a computational model for subjective value. For choice data, we evaluated the impact of image type by calculating the proportion of times a given participant chose the ambiguous lottery for emotional vs neutral images. If synchronized affect increases ambiguity aversion, we would expect to find that participants would be less likely to select ambiguous lotteries paired with emotionally evocative images. Additionally, we implemented a computational model to obtain a more fine grain metric for the effect.

We used a model for participants' subjective value of the choice options (Gilboa &

Schmeidler, 1989).

$$SV(p, A, v) = \left(p - \beta\frac{A}{2}\right) \times v^{\alpha}$$

1

where, *p* corresponds to the probability of winning amount *v* for that lottery, and *A* corresponds to the proportion of the distribution covered by the occluder. All ambiguous lotteries set $p = .5$. $\alpha$ represents a person's attitudes toward risk with values less than one indicating risk aversion and values greater than 1 indicating risk seeking behavior. $\alpha$ is asymmetric about 1, such that a value of .5 is equally as averse to risk as a value of 2 is risk seeking. Alternatively, $\beta$ represents a person's ambiguity preferences with positive values corresponding to ambiguity aversion and negative values corresponding to the opposite. Unlike $\alpha$, $\beta$ is symmetric, meaning the value −1 and 1 indicate the same magnitude of preference while the sign indicates the direction.

Subjective value estimates were compared to the reference option (a certain $5).

The probability of choosing the lotteries were calculated as the inverse logit of the difference between the subjective values (Eq. 2), and free parameters where fit using maximum likelihood estimation (Luce, 1959) in Python using the SciPy package (Virtanen et al., 2020).

$$p\left(ChooseLottery\right) = \frac{1}{1 + e^{\gamma(SV_R - SV_L)}}$$

2

$\beta$ estimates across image types were compared to assess the impact of image type of ambiguity preferences. If high arousing images increased ambiguity aversion, we would expect to see significantly higher estimates for lotteries paired with emotionally evocative images.

## Data Preparation and Screening

Due to the inherent problems with data quality for online studies, we implemented several data quality assessments. First, there were a series of three comprehension check questions to ensure participants understood the task; participants that failed the comprehension check were redirected back to Prolific and were not allowed to participate. We also implemented attention checks during the survey. Throughout the Qualtrics survey they were asked questions to probe whether or not they were attending to the survey questions (e.g., "how many *fatal* heart attacks you had in that last year?"). Participants that failed to accurately answer attention check questions were excluded from analyzes ($N = 1$). We also assessed the quality of participants’ choice data. Specifically, we excluded participants with low or no variance (more than 97% choices were the safe option) for their ambiguous lottery choices ($N = 33$).

Finally, the computational model parameter estimates had additional screening procedures. Participants that did not pass these additional screenings were not included in analyses of the model’s parameters but were included for analyses of the raw choice data.

Those with poor model fit (Pseudo $R^2 < .3$; $N = 14$) were excluded from model analyses. Furthermore, parameter estimates of the computational model were constrained. The trials’ parameters (i.e., win amounts and risk/ambiguity levels) used for the experiment only covered a subset of the parameter space; therefore, they can only meaningfully estimate parameters inside that space ($.11 > \alpha > 7.6$ and $-3.9 > \beta > 4.16$). These parameter constraints were analytically derived directly from the edges of the trial space. Participants were excluded if their parameters hit these bounds ($N = 7$) indicating their preferences could not be properly estimated from the set of offered choices.

### Results

To ensure the manipulation was successful, we ran several paired samples t-tests on the affect ratings comparing high and low arousing images. As predicted, there were large, significant differences in affect ratings for the two groups of images. Specifically, we found that participants rated the emotionally evocative images as more arousing, $t(165) = 25.965$, $p < .001$, $d = 2.705$ 95% CI [2.262, 3.147], and more negatively valenced, $t(165) = 27.645$, $p < .001$, $d = 3.319$ 95% CI [2.712, 3.926], compared to the neutral images (see Fig. 2).

## Behavioral Choice Data

To assess the impact of image type on uncertainty preferences we used orthogonal contrasts. The first contrast compared the two uncertainty types and the second evaluated the impact of image type on participants’ ambiguity preferences. In line with previous literature, we found that subjects were relatively more ambiguity averse than risk averse, $t(166) = -5.729$, $p < .001$, $d = -0.310$ 95% CI [-0.414, -0.207].

Furthermore, we found that synchronized, emotionally evocative images increased ambiguity aversion $t(166) = -2.038$, $p = 0.043$, $d = -0.034$ 95% CI [-0.068, -0.001] relative to neutral images (see Fig. 3a). In other words, participants were less likely to choose an ambiguous lottery when it was paired with an emotionally charged image, compared to a neutral image.

## Computational Model Parameter Estimates

In line with non-parametric assessment above, we found that $\beta$ estimates were significantly greater than zero indicating clear evidence of ambiguity aversion, $t(145) = 7.859$, $p < .001$, $d = 0.650$ 95% CI [0.348, 1.026]. More importantly, the analysis indicated that high arousal images increased ambiguity aversion relative to neutral images, $t(145) = 2.111$, $p = 0.037$, $d = 0.073$ 95% CI [0.005, 0.141] (see Fig. 3b).

To assess the nature of this relationship we explored whether general ambiguity preferences moderated the magnitude that one's ambiguity preferences shifted from emotionally evocative images. We calculated the difference in probability of choosing an ambiguous lottery when paired with a neutral compared to an emotionally evocative image. This difference score was then regressed onto participants' ambiguity attitudes ($\beta$). We specifically used $\beta$ estimates calculated from the neutral context, as this should serve as their baseline attitudes towards ambiguity. The effects of emotional evocative images on ambiguity choices were consistent across the range of ambiguity attitudes, $t(144) = -1.547$, $p = 0.124$, *b*-coef = -0.0155 95% [-0.0353, 0.0043].

We then assessed if the degree to which participants are impacted by emotionally evocative images could be related to how uncertain a participant was when making a decision. One additional feature of the computational model is that it provides predicted probabilities for choosing the lottery, which assumes a sigmoid shape. Therefore, the model predicts that for some lotteries it is very clear that the participant will choose the lottery

(e.g., a chance of winning $66 with only 24% of the distribution occluded) or that they will choose the certain $5 option (e.g., a chance of only winning $6 with 74% of the distribution occluded). By extension, this also means that there are a subset of trials which offer options with a subjective value close to $5 for a given participant on which they are less certain and may be more subject to change as a result of the incidental affect manipulation. To test whether choice certainty affected this relationship, we regressed choice probability for ambiguous lotteries on the model predicted probability, image condition, and the interaction. Again, we used the predicted choice probability estimated based on their baseline attitudes calculated from the control trials. Critically, there was a significant interaction, $z = 3.687$, *b*-coef = -0.755 (0.205), $p < .001$. We found that as the predicted probability of choosing the lottery increased, participants were more likely to be influenced by an emotionally evocative image (see Fig. 4). In other words, participants' choices were only affected by emotional evocative images when they considered choosing the lottery. Finally, to ensure these effects were specific to changes in ambiguity attitudes as opposed to increasing decision noise we compared the noise term ($\gamma$) across the two conditions. As predicted, there were no differences in decision noise across the two conditions, t(145) = -1.467, p = 0.144, d = -0.128 95%

CI [-0.286, 0.031], indicating the effect of emotionally evocative images was specific to participant's ambiguity preferences and did not result from an increase in the stochasticity of their choices.

## Individual Differences

Participants who rated the emotionally evocative images as more extreme compared to neutral images might also be expected to have larger changes in their ambiguity choices. To test this hypothesis, we calculated the difference in valence and arousal ratings for the two groups of images (neutral vs. high arousal) that participants viewed during the lottery task. These differences in perceived affect were then used to predict the difference in the probability of choosing the lottery paired with the two image types. Neither valence nor arousal difference scores predicted linear changes in ambiguity preferences (all $p$'s $> .05$). Thus, although there were differences in the probability of choosing an ambiguous lottery across the two types of images, it appears that this is not a linear effect (e.g., larger differences in affect ratings are not associated with larger differences in choices).

In addition to subjective affect ratings, we also evaluated whether the psychological traits measured by survey questionnaires predicted ambiguity preferences. Due to concerns for multicollinearity, we ran a series of correlations and corrected for multiple comparisons. Consistent with previous research (Sambrano et al., 2022), we found no relationship between ambiguity preferences in the neutral image condition and intolerance of uncertainty ($r(164) = 0.042$, $p = 0.592$), positive affect ($r(164) = -0.041$, $p = 0.601$), perceived stress ($r(164) = -0.010$, $p = 0.900$), or trait anxiety ($r(164) = 0.012$, $p = 0.875$). Of note, negative affect was significant at the .05 level, but did not survive correction for multiple comparisons, $r(164) = -0.174$, $p = 0.025$.

Although we did not find any relationship between these questionnaires and ambiguity preferences in general, we did find some relationships between these questionnaires and the change in ambiguity aversion as a result of the incidental affect manipulation. Specifically, we found that the intolerance of uncertainty scale (IOS) was positively correlated with the difference in probability of choosing the lottery across image types, $r(164) = 0.213$, $p = 0.006$. As participants were *less* tolerant of uncertainty, they also tended to be more influenced by emotionally evocative images paired with ambiguous lotteries. Similarly, trait anxiety was also positively correlated with changes in ambiguity preferences, such that higher rates of trait anxiety were also associated with greater changes in ambiguity attitudes as a result of the emotionally evocative images, $r(164) = 0.190$, $p = 0.014$. In other words, the ambiguity preferences of those with high intolerance of uncertainty and high trait anxiety tended to be influenced more strongly by emotional images. Scores on the Positive/Negative Affect Scale and the Perceived Stress Scale were uncorrelated with the change in ambiguity preferences (all $p$'s $> .07$ uncorrected).

### Discussion

This study aimed to evaluate the circumstances under which incidental affect can alter decisions under uncertainty. Specifically, we tested whether synchronizing an emotionally evocative image with the occluder of an ambiguous lottery can increase ambiguity aversion. The data indicated that synchronizing emotionally evocative images led to a small, but statistically significant, increase in ambiguity aversion in lottery choices. Additionally, we found this effect was primarily driven by a subset of trials. Participants' choices were most affected by synchronized emotional images when they considered choosing the ambiguous lottery. Choices where it was clear that the participant would choose the guaranteed option based on their pre-existing preferences $5 (e.g., a chance of only winning $6 with 74% of the distribution occluded) were relatively unaffected by the manipulation. Finally, trait anxiety and intolerance of uncertainty predicted the degree to which participants' ambiguity preferences shifted as a result of the incidental affect manipulation.

Presenting emotionally evocative images synchronized with choices increased ambiguity aversion. While previous studies have shown a link between integral affect and ambiguity aversion (FeldmanHall et al., 2016; Levy et al., 2010), this is the first study to alter these preferences through an acute, incidental affect manipulation. Despite threats of shock and the Trier Social Stress Test being more potent affect manipulations, previous attempts were unsuccessful (Sambrano et al., 2022). We hypothesize that this was because, in the present study, the images were synchronized to the choice onset making it more difficult to dissociate the choice drive and image driven affective responses. As a result, the arousal induced by the images was at least partially, non optimally included in the lottery's utility calculation; ultimately resulting in a shift to increase ambiguity aversion. This is consistent with previous research that showed that when people are aware of the source of their incidental affective response, it is less likely to affect their judgements (Schachter & Singer, 1962; Schwarz & Clore, 1983).

One might suspect that the change in ambiguity attitudes could be due to a Pavlovian instrumental transfer (PIT), where the effect of the negative valence of the images are transferred to the ambiguous lotteries. Although PIT could contribute to the overall effect, we hypothesize that it alone is not sufficient to explain the present findings. In a typical PIT experiment, a conditioned stimulus (e.g., an image) is paired with a rewarding stimulus (e.g., food). Then the subject learns to associate a behavior (e.g., button press) with the reward in the absence of the conditioned stimulus. In a final assessment, PIT is said to occur if the rewarding behavior (e.g., button press) is performed more in the presence of the conditioned stimulus (e.g., image) than without. Importantly, none of the incidental affect studies on ambiguity aversion have used Pavlovian conditioning specifically, but the primary proposed mechanism for PIT is that the affect/value of a stimulus can be transferred to a behavior. Critically, this could have occurred either in the present study or in Sambrano et al. (2022), in which there was no effect. If the affective experiences were transferred to ambiguity preferences, we would expect that threats of shock used by Sambrano et al. (2022) would have also affected these choices, since this manipulation closely matched typical PIT experiments. Specifically, the shocks were paired with one colored background and a second background color signaled safety. If PIT underlies the impact of incidental affect on ambiguity aversion, we would have expected that the negative affect associated with the colored background paired with shocks would have increased ambiguity aversion relative to trials using a background not paired with shocks. Nonetheless, threats of shock were unsuccessful in changing ambiguity preferences in Sambrano et al. (2022). Given this, we believe that it is unlikely that PIT underlies the impact of incidental affect on ambiguity aversion in the present study, and hypothesize that, at least in part, the arousal of the image was unintentionally incorporated into the lottery's utility.

Overall people were affected by emotionally evocative images in the present study, but they were more likely to be affected when, as predicted by the model, they considered choosing the lottery as opposed to the reference option. One possible explanation for this finding is that people are likely to attend more to lotteries they are considering choosing. When participants considered choosing the lottery option, they likely also spent more time viewing the lottery, and by extension the occluding image; allowing it to unintentionally incorporate into the utility function. Future studies could monitor eye gaze while synchronizing images to test this hypothesis.

Consistent with Sambrano et al. (2022), we found that trait anxiety (TA) and intolerance of uncertainty (IoU) were not correlated with ambiguity preferences in general. Interestingly, we found that TA and IoU were associated with how much ambiguity attitudes were affected by emotional images. Specifically, the higher one's TA and IoU the more they were affected by synchronized, incidental affect, such that participants became relatively more averse to ambiguity when choices were paired with negative arousing images, compared to those lower on TA and IoU. This finding is consistent with the rich history of research on attention and affect. For instance, high TA is associated with an attentional bias towards negatively valenced stimuli (for a meta-analytic review see Bar-Haim et al., 2007), and this attentional bias can also lead to changes in perception (Barbot & Carrasco, 2018; Ferneyhough et al., 2013). Individuals high on the IoU scale show similar attentional biases to negatively valenced images (Morriss & McSorley, 2019; Morriss et al., 2018). In the context of the present study, those with high TA and IoU might have had more difficulties disengaging from the negatively valenced images while making choices. As a result, these individuals may have been more likely to incorporate the image evoked affective response into the lotteries utility calculation leading to increased ambiguity aversion.

Research has shown that the relationship between affect and decision making is complex. It is increasingly clear that there is not one singular way in which affect can impact our decisions. The impact of incidental affect on decisions can be subtle and change based on the context of how the incidental affect was evoked or the personal characteristics and history of the individual. By understanding the complex relationship between affect and decision making we can identify how affect may incidentally bias our decisions, which is the first step necessary to correct these unintended biases and improve our decisions.

## Declarations

## DATA AVAILABILITY STATEMENT

The data supporting this study’s findings are available on Open Science Framework: https://osf.io/smu5w/.

**FUNDING STATEMENT**

This research was funded by the National Institutes of Health, R01-DA042855.

**ETHICS STATEMENT**

The study followed ethical guidelines outlined by the APA and approval was received by the Institutional Review Board at Harvard University.

## Figures

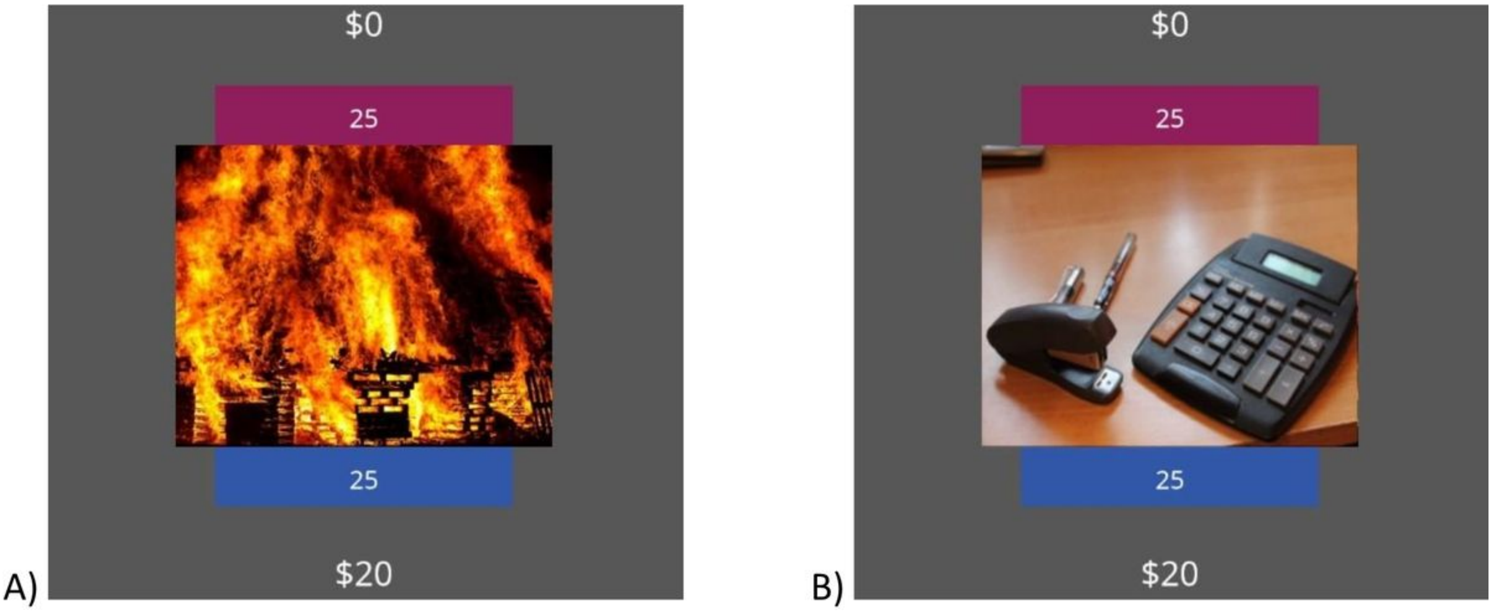

**Figure 1**

*The occluder was replaced with an (A) emotionally evocative or (B) neutral image. The images elicited an affective response that is expected to be non-optimally incorporated into the value estimate for the choice options.*

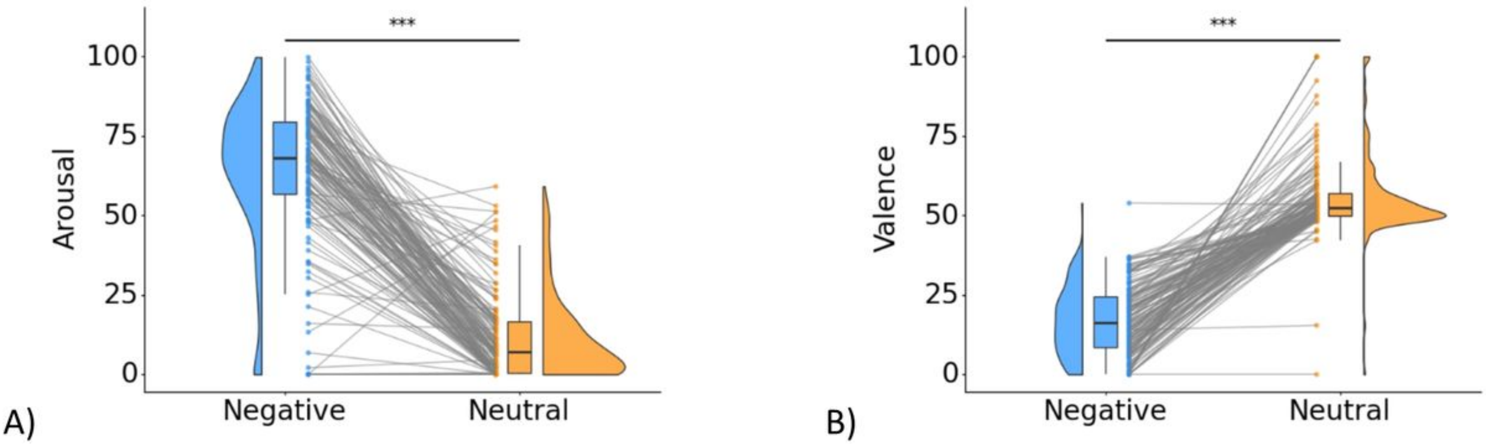

**Figure 2**

*Affect rating by image type for (A) arousal and (B) valence. Participants rated emotional evocative images as more arousing and more negatively valenced compared to neutral images indicating a successful manipulation.*

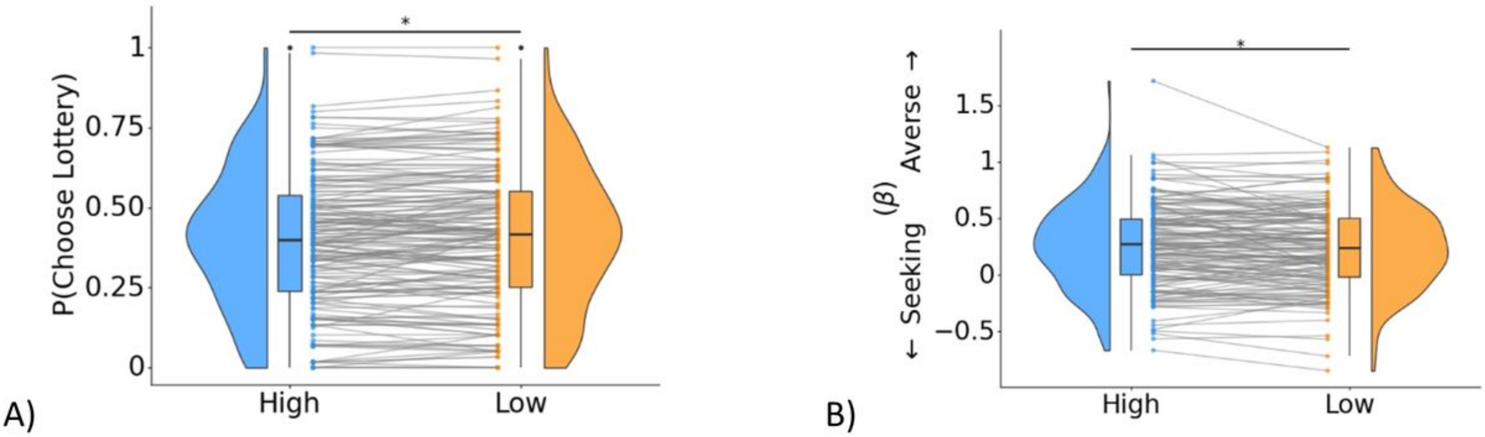

### Figure 3

*Ambiguity preferences were affected by synchronized, negatively-valenced, high arousal images compared to neutral images. These differences were seen in both (A) raw choice data and (B) computational model parameter estimates.*

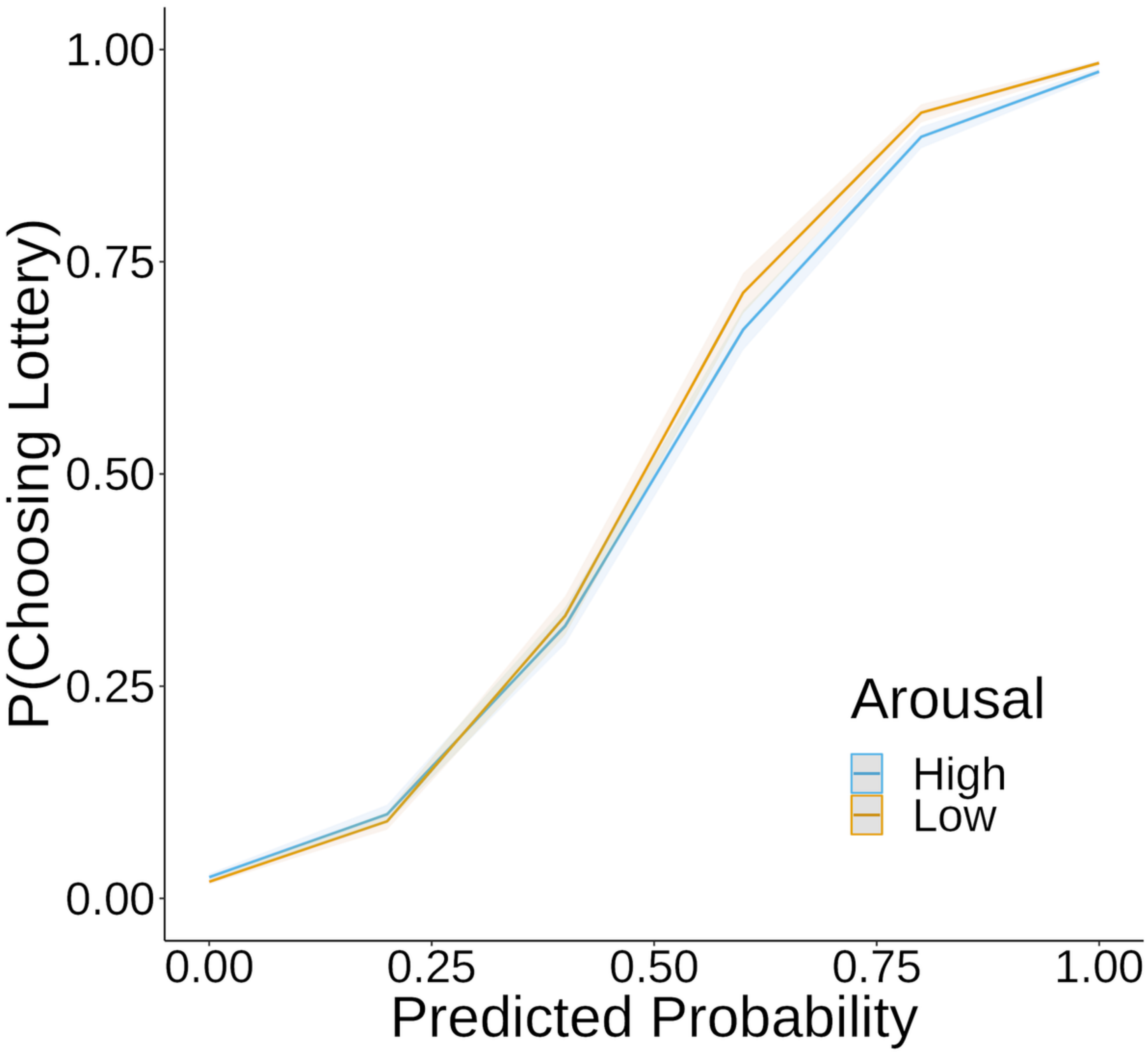

**Figure 4**

*Ambiguity choices were most affected by emotional images when the model predicted that participants would choose the lottery option as opposed to when it predicted that participants would choose the certain option.*

### Supplementary Files

This is a list of supplementary files associated with this preprint. Click to download.

- Appendix.docx
